# Supplementary material for: Patterns of Intron Gain and Loss in Fungi
Source: PLoS Biol. 2004 Nov 30;2(12):e422. doi: 10.1371/journal.pbio.0020422 (PMC532390; doi:10.1371/journal.pbio.0020422)
Supplement: Table S1 — Also available at http://genes.mit.edu/NielsenEtAl/. (4.3 MB ZIP). [file pbio.0020422.st001.zip › NielsenEtAl/html/606.html]

AN4279.1.NCU02814.1.MG01596.1.FG07121.1


```
 CLUSTAL W (1.82) Multiple Sequence Alignments - Introns Inserted


Sequence 1: NCU02814.1	712 aa
Sequence 2: MG01596.1	674 aa
Sequence 3: FG07121.1	672 aa
Sequence 4: AN4279.1	648 aa
Alignment Length: 749 aa
Number Identitical Residues: 292 aa
Alignment Score (without introns) 14350


MG01596.1 	---------------~---MVPRAEGS-QLKRAR0PSTADSDGESFKKPRRTERAAHKAE
NCU02814.1	---------------~---MAPRAEGSSTLKRAR0GSTDDND--SFKKPRRSERLSSQRT
FG07121.1 	---------------~---MAPQPEKS-QLKRQR0GSLPEDE-TETKKPRRTERLAVDAD
AN4279.1  	MELESSAFPKTLAEQ2DNTMVPRNEQS-SLKRSR0-VTADHESQDQKKPRRSERISSQQQ
          	 . .::: ..: :.. ..:*.*: * *  *** *     : :. . *****:** : .  

MG01596.1 	ESTAQP--KTPVDTKQQLPSPITHNPTEGSFDAYKEGTVTPPEGRPSQVAHRSP-VQGFS
NCU02814.1	GDSPEPKQKTPLKTTKQLPSPVTHDSDEFPQDS-KEPTATPPAGRPSQLSQISPRPENQH
FG07121.1 	--------KTPVVNKQHLPSPLTHLTDD-SSELNKDSTVTPPEKKETESTPKKS-EDNSQ
AN4279.1  	S-------QTPVIQS-YLPTPLTHHDST-ATDLRNEMTATPP----SQALPRSP------
          	        :**:  .  **:*:**     . :  :: *.***    ::    ..      

MG01596.1 	SPPQDTQATQAIP-SQIVDPKAALSDEVEDEVKEGVWGYLFPLDTRYGGKCVVLRKRGSC
NCU02814.1	SQSQASQLNDTQAFSQLPDLDKALSDEVEDEVKEGVWGYLFPLDPRYGGRCVVLRRRAAC
FG07121.1 	SQALSSPPQDTQPLSQFVDRHSAISDDIEDEIREGVWGYLVPLDPKYGDKPIVLKKRSAC
AN4279.1  	-SDLGSPPGDTQALSQFVYPPRAFADDVEDEAAEGVWGYLIPLDDKVR-DALVLRKRDGC
          	     :   :: . **:     *::*::***  *******.*** :     :**::* .*

MG01596.1 	PMPDTN-------------GKSDDKPCDKASK------------------------D1IV
NCU02814.1	PLPDTVSQAV-----GSKRGKRGQKALIKEEHDLDKTK-VKGLPSGGYLIGRHPECD1IQ
FG07121.1 	PLPDTVSDAAKKENKNNTNDKNGKSAAMKDEETFEKKKKEKGAPSGGYLIGRHPECD1IV
AN4279.1  	DAKPTKERSS-------KERKSRESSTKHAANR----------RPGGYLIGRHPECD1LS
          	    * . :        .  *  ...  :  .            ...   .  ...* : 

MG01596.1 	VDDPIVSNRHCLIFAEHKGNDTVVILEDLSSNGTFVNEAIVGRNQRRELLEQDEIAVKDT
NCU02814.1	IEDPIVSNRHCIIFTENKGNDTIAVLEDLSSNGTFVNEAIVGRNRRRELQELDEIAVLGT
FG07121.1 	VNDGIVSNRHCLLFTENKGNDTVAIVEDLSSNGTYVNEAIVGRNQRRELEEQDEIAVHGK
AN4279.1  	LNIPTVSNRHFLVFPENRRGDTVAILEDLSSNGTFINDAIVGRNKHRELEDGDEVTVLDE
          	::   ***** ::*.*:: .**:.::********::*:******::*** : **::* . 

MG01596.1 	ARFIFRYPKYRQTSAFSQQYTLLGKLGKGHFAEVFACVEKSTGQRYAVKIFSMVPGLEER
NCU02814.1	ARFIFRYPKSRHTSAFRQQYTMLQKLGKGHFAEVYLCVEKSTGTQYAVKVFSKTPGVEER
FG07121.1 	ARFVFRYPQTRQTSAFLQQYTLLEKLGKGHFAEVYLCVEKTTGQRFAVKIFTKHPGVEDR
AN4279.1  	VRFVFRAPRTKNISGFRQQYRVLQQLGKGHFATVYLCVERTTGAKFAVKVFEKR--ADSK
          	.**:** *: :: *.* *** :* :******* *: ***::** ::***:*      :.:

MG01596.1 	SRTEGLQLEIAVLMGVSHPNVLCLKDTFHEGNAVYLVLELAPEGELFNFIVMKQKLSEAE
NCU02814.1	SKDEGLQQEIAVLMGVSHPNVLCLKDTFNEPNAVHLVLELAPGGELFNYIVKKTKLSENE
FG07121.1 	SKTEGLQQEIGVLMGVSHPNVLCLKDTFNERDRVYLVLELAPEGELFNFIVMKQKLSEDE
AN4279.1  	SQNDALMQEIGLLMSVSHPNLLCLKDTFDESDGVYLVLELAPEGELFNMIIRKQKFTEKE
          	*: :.*  **.:**.*****:*******.* : *:******* ***** *: * *::* *

MG01596.1 	SRKLFTQLFHGVKYL0HDRNIVHRDIKPENILLVDKDLHVKIADFGLAKIIGEESFTTSL
NCU02814.1	CRKLFTQLFQGVKYL~HDRNIVHRDIKPENILLVDDDLHVKLADFGLAKIIGEESFTTTL
FG07121.1 	SRKLFVQLFQGIKYL0HERNIVHRDIKPENILLVDKNLHVKLADFGLAKIIGEESFTTTL
AN4279.1  	TRHIFRQLFEGLKYL0HDRGIVHRDIKPENILVADNKLTVKLGDFGLAKIIGEDSFTTTL
          	 *::* ***.*:*** *:*.************:.*..* **:.**********:****:*

MG01596.1 	~CGTPSY~VAPEILVDGRHRKYTKAVDVWSLGVVLYICLCGFPPFSDELTSESFPYTLTQ
NCU02814.1	~CGTPSY~VAPEILTDTRHRKYTKAVDVWSLGVVLYICLCGFPPFSDELTSPDFPYSLSD
FG07121.1 	~CGTPSY~VAPEILADSKQRKYTKAVDVWSLGVVLYICLCGFPPFSDELYSRDFPFTLSQ
AN4279.1  	2CGTPSY1VAPEILQESRRRKYTKAVDIWSLGVVLYICLCGFPPFSDELYTPENPYTLAQ
          	 ****** ****** : ::********:********************* : . *::*::

MG01596.1 	QIKNGIFHYPSPYWDSVGDPAL1SLIDCMLVVDPEKRYTIDQCLAHPWMTQAQPSVHDST
NCU02814.1	QIRQGKFDYPSPYWDPVDDLAL1DLIDSMLVVDPEKRFTIDDCLSHPWMTQKTPGVNDST
FG07121.1 	QIKSGRFDYPSPYWDSVGDPAL1DLIDSMLIVDPEKRYTIDQCLQHPWLTQSSPSVNDST
AN4279.1  	QIKLGRFDYPSPYWDSVGDPAL~DLIDRMLTVDVDKRITVDECLQHPWLTGELPSVTDST
          	**: * *.*******.*.* ** .*** ** ** :** *:*:** ***:*   *.* ***

MG01596.1 	DGLVGGFAGLEVNRRGIVRERTLLSSINSVQVT-RVPAGPNQEPVKVFIKNPKD------
NCU02814.1	NGLVNGIAGLDVTRRGVLRERTLLSSINTVEIADKIPLGENKPELKIYKKNPTETVGGPS
FG07121.1 	GGLVGGIASLQVQRRAAARERTLLSSLNTVEVTAQLDAGKDKRPVKVFAKNKGR------
AN4279.1  	DGLTGALGNLDFSKRKIARERTLLSGINDVRSSQQKTG--DGASVKVYHKNIAG------
          	.**...:..*:. :*   *******.:* *. : :     :   :*:: **         

MG01596.1 	--------GKANRAPEPGPSQNRRPDEFIEMGGKGDQPLFGHDGGSNYPSADIASQKTAI
NCU02814.1	EHELAGAQAGPSRQKEARPDDNRDPNEFMKMGGKGDEVLFADDPKSNYPTAKKDAKDIVE
FG07121.1 	---------VTNFSKESDPASRRAPDEFIEMGGKGDQELFPDDDSSIYPTGDNGEKIKAT
AN4279.1  	----QRMHNQRATRQHEFPNKNSGPKDFVNLGECGDPVLFEEEPTSRHN-----------
          	               .  * ..  *.:*:::*  **  ** .:  * :            

MG01596.1 	SQPALNGAE~TSKDDEVKEINANGKTKPTAAGKGKEH
NCU02814.1	STTKTNGKG1-------KLASGFGIVIMDARFHG---
FG07121.1 	KAQR-----~---------------------------
AN4279.1  	---------~---------------------------
          	
```
